# Study of Thermal Analysis Behavior of Fenbendazole and Rafoxanide

**DOI:** 10.15171/apb.2017.039

**Published:** 2017-06-30

**Authors:** Ali Kamal Attia, Ahmed Sayed Saad, Manal Sami Alaraki, Eman Saad Elzanfaly

**Affiliations:** ^1^National Organization for Drug Control and Research, P.O. Box 29, Cairo, Egypt.; ^2^Analytical Chemistry Department, Faculty of Pharmacy, Cairo University, Kasr El-Aini Street, Cairo, Egypt.

**Keywords:** Fenbendazole, Rafoxanide, Thermal analysis, Purity, Molecular orbital calculations

## Abstract

***Purpose:*** Thermal analysis techniques have been applied to study the thermal behavior of fenbendazole (Fen) and rafoxanide (Raf). Semi-empirical molecular orbital calculations were used to confirm these results.

***Methods:*** Thermogravimetric analysis, derivative thermogravimetry, differential thermal analysis and differential scanning calorimetry were used to determine the thermal behavior and purity of the drugs under investigation.

***Results:*** Thermal behavior of Fen and Raf were augmented using semi-empirical molecular orbital calculations. The purity values were found to be 99.17% and 99.60% for Fen and Raf, respectively.

***Conclusion:*** Thermal analysis techniques gave satisfactory results to obtain quality control parameters such as melting point and degree of purity at low cost, furthermore, its simplicity and sensitivity justifies its application in quality control laboratories.

## Introduction

Fen is chemically named as methyl [5-(phenylsulfanyl)-1H-benzoimidazol-2-yl] carbamate.^1^ It is a benzimidazole carbamate anthelmintic utilized in veterinary medicine as antiparasitic drug.^2^

Raf is chemically named as N-[3-chloro-4-(4-chlorophenoxy) phenyl]-2-hydroxy-3,5-diiodo-benzamide.^3^ It is an anthelmintic used in veterinary medicine for the treatment of fascioliasis in cattle and sheep.^2^

Fen and Raf are co-formulated as oral suspension which is widely used as a broad spectrum anthelmintic.

Thermal analysis methods such as thermogravimetric analysis (TGA), derivative thermogravimetric analysis (DrTGA), differential thermal analysis (DTA) and differential scanning calorimetry (DSC) find wide spread use for both quality control and research applications on industrial products, such as polymers and pharmaceuticals.^4-6^ Thermal analysis has great importance in characterization, polymorphism identification, determination of activation energy values and purity evaluation of drugs.^7^

The present work is the first attempt to study the thermal behavior of Fen and Raf and to determine their purities. Semi-empirical molecular orbital (MO) calculations were used to determine the weakest bonds ruptured during thermal degradation of the drugs to confirm thermal analysis results.^8^

## Materials and Methods

### Materials 

Fen and Raf were obtained from Pharma-Swede, Egypt. The purity values were 99.00%^1^ and 99.40% using potentiometric titration,^1^ and spectrophotometric methods,^9^ for Fen and Raf, respectively. 

### Instruments and Methods

- Shimadzu thermogravimetric analyzer DTG-60 H with TA 60 software (Tokyo – Japan) was used; aluminum oxide is used as a reference. The experiments were achieved in platinum crucible up to 800 °C at different heating rates (5, 10, 15 and 20 °C min^-1^) in dry nitrogen atmosphere (flow rate of 30 ml min^-1^). About 5 mg of drug was used. The activation energy of decomposition (*E*) was calculated using Ozawa’s method.^10^
- Shimadzu-DSC 50 (Tokyo – Japan) was used to obtain the DSC curves of Fen and Raf up to 350 °C at heating rate of 10 °C min^-1^ in dry nitrogen atmosphere (flow rate of 30 ml min^-1^). About 3.0 mg of drug was putted in an aluminum pan. An empty aluminum pan was used as a reference.
- Shimadzu-GC–MS-QP 1000 EX quadruple mass spectrometer was used to obtain the mass spectra of Fen and Raf.
- MO calculations were done using ChemBio3D Ultra 2010 (MM2 minimization method).^11,12^

## Results and Discussion

### Thermal analysis 

**Figure 1:**
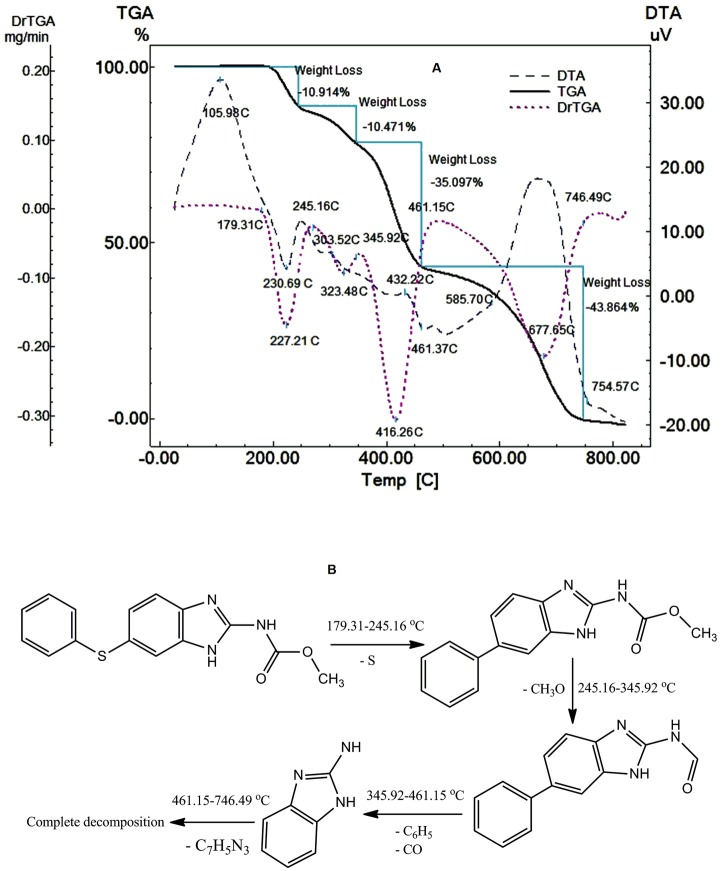
Thermal analysis curves of Fen at heating rate of 10 °C min^-1^ in dynamic nitrogen atmosphere with the flow rate of 30 mL min^-1^ (A). The thermal degradation mechanism of Fen (B).

Fen decomposes in four steps; the first step starts at179.31 °C up to 245.16 °C through the loss of sulfur atom^13^ (Found 10.914%, Clac. 10.688%) showing exothermic peak at 105.98°C (glass transition) and endothermic peak at 230.69 °C which may be due to melting of Fen, it melts with decomposition. The drug continues to decompose in the second step from 245.16 °C to 345.92 °C due to the loss of CH_3_O (Found 10.47%, Clac. 10.35%) showing weak exothermic peak at 303.52 °C. In the third step it decomposes from 345.4 °C to 461.15 °C due to the loss of C_6_H_5_ and CO molecules (Found 35.097%, Clac. 35.07%) showing weak exothermic peak at 432.22 °C. In the last step it decomposes from 461.15 °C to 746.49 °C due to the loss of C_7_H_5_N_3_ molecule (Found 43.86%, Clac. 43.75%) showing broad exothermic peak between 585.70°C and 754.57 °C (Figure 1). Figure 1 shows the thermal degradation mechanism of Fen.

**Figure 2:**
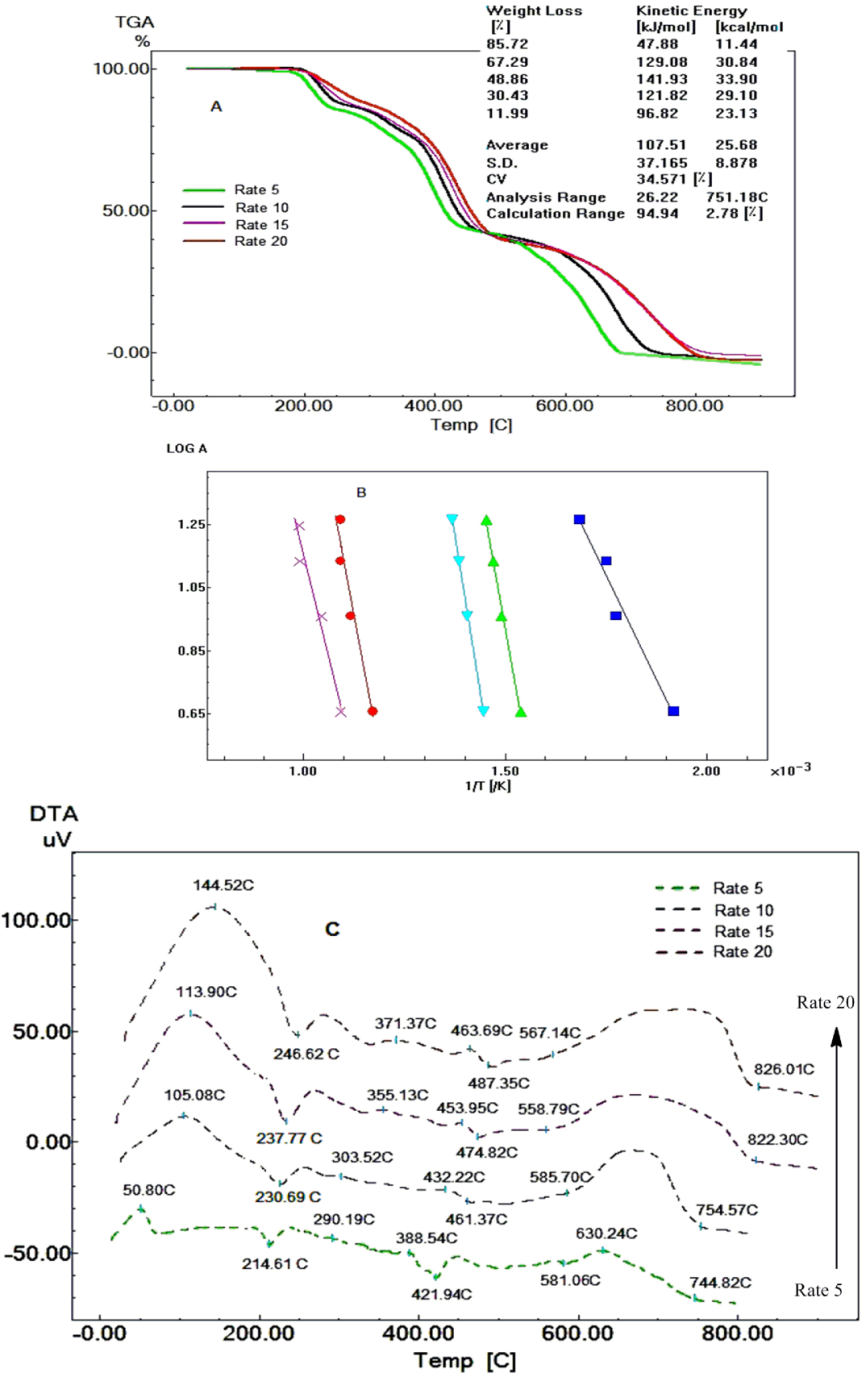
TGA curves of Fen at different heating rates under dynamic nitrogen atmosphere (A). Ozawa’s plot of Fen with correlation of the curves presenting a linear tendency (B). DTA peaks of Fen at different heating rates (C).

Figure 2A and 2C show the TGA and DTA curves of Fen at different heating rates (5, 10, 15 and 20 °C min^-1^); these curves are shifted to higher temperatures when the heating rate increases. Figure 2B shows linear relations (Ozawa's plot).

**Figure 3:**
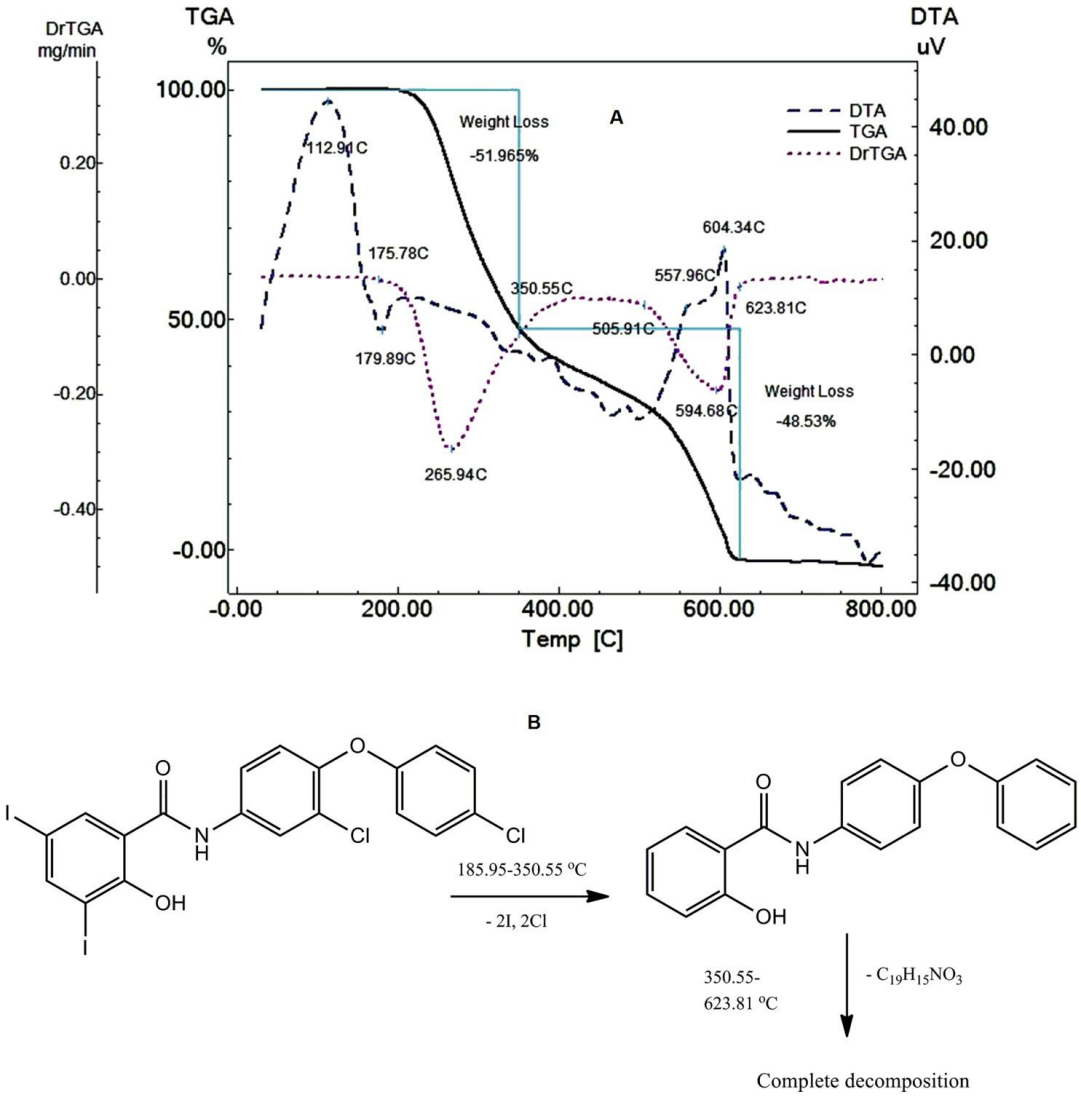
Thermal analysis curves of Raf at heating rate of 10 °C min^-1^ in dynamic nitrogen atmosphere with the flow rate of 30 mL min^-1^ (A). The thermal degradation mechanism of Raf (B).

Figure 3 shows that Raf decomposes in two steps; the first step starts at 185.95 ^o^C up to 350.55 °C through the loss of two iodine and two chlorine atoms (Found 51.965%, Clac. 51.885%) showing exothermic peak at 112.91°C and endothermic peak at 173.88 °C which may be attributed to glass transition and melting of Raf, respectively. The drug continues to decompose in the second step from 350.55 oC to 623.81 °C due to the loss of C_19_H_15_NO_3_ (Found 48.53%, Clac.48.12%) showing two exothermic peaks at 557.96 °C (broad peak) and 604.34 °C (sharp peak). Figure 3 shows the thermal degradation mechanism of Raf.

**Figure 4:**
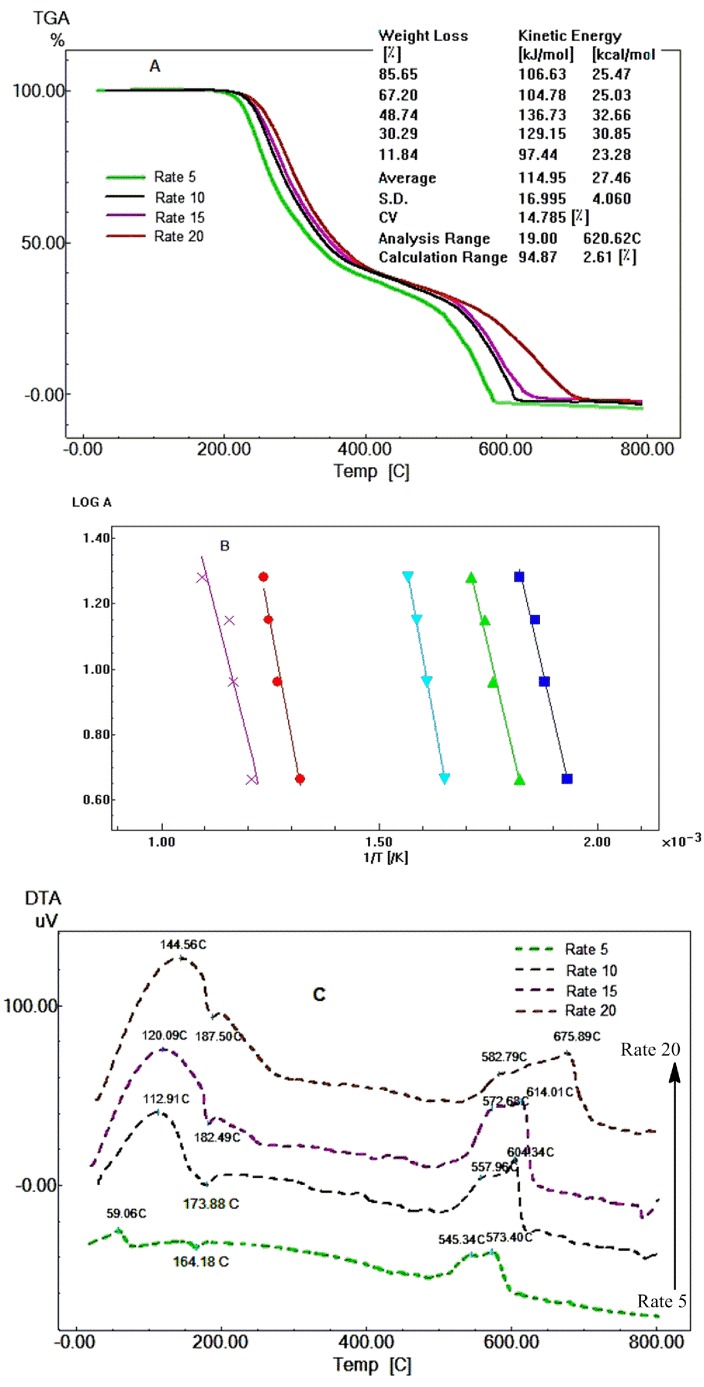
TGA curves of Raf at different heating rates under dynamic nitrogen atmosphere (A). Ozawa’s plot of Raf with correlation of the curves presenting a linear tendency (B). DTA peaks of Raf at different heating rates (C).

Figure 4A and 4C show that the TGA and DTA curves of Raf are shifted to higher temperature values as the heating rate increases. Figure 4B shows linear Ozawa’s plot.

*E* values of Fen (107.51 KJ mol^-1^) and Raf (114.95 KJ mol^-1^), therefore, Raf is relatively more thermally stable than Fen and these results are compatible with their thermal decomposition curves, where Fen starts to decompose at 179.31°C and Raf starts its decomposition at 185.95 °C.

### MO calculations

Electronic Supplementary Information 1 (ESI 1) shows the numbering of atoms of Fen and Raf. ESI 2 shows the bond lengths of different bonds of the drugs.

For Fen, the loss of sulfur atom is due to the rupture of S(7)-C(8) and C(4)-S(7) bonds (first step), the loss of CH_3_O is due to the rupture of O(19)-C(20) bond (second step), the loss of C_6_H_5_ and CO molecules is due to the rupture of N(17)-C(18) and C(4)-C(8) (third step), in the fourth step the loss of C_7_H_5_N_3_ is due to the rupture of C(18)-O(19), C(8)-C(13), C(9)-C(8), C(13)-C(12), C(15)-N(17), C(4)-C(5), C(3)-C(4), C(1)-C(6), C(5)-C(6), C(2)-C(3), C(1)-C(2), C(10)-C(11), C(12)-C(11), C(10)-C(9), N(16)-C(15), C(15)-N(14), N(16)-C(11), N(14)-C(10) and C(18)-O(21) bonds, respectively according to their bond lengths (ESI 2).

For Raf, the loss of two iodine atoms is due to the rupture of C(1)-I(7) and C(3)-I(8) bonds and the loss of two chlorine atoms is due to the rupture of C(17)-Cl(19) and C(24)-Cl(27) bonds for the first decomposition step of Raf. The loss of C_19_H_15_NO_3_ in the second decomposition step includes the rupture of C(16)-O(20), O(20)-C(21), C(5)-C(10), C(6)-O(9), C(10)-N(11), C(5)-C(6), C(16)-C(17), C(4)-C(5), N(11)-C(13), C(1)-C(6), C(21)-C(26), C(21)-C(22), C(17)-C(18), C(15)-C(16), C(13)-C(18), C(13)-C(14), C(25)-C(26), C(24)-C(25), C(22)-C(23), C(1)-C(2), C(14)-C(15) and C(2)-C(3) bonds, respectively according to their bond lengths (ESI 2).

ESI 3 shows the mass spectra of Fen and Raf. The results indicate the compatibility between thermal degradation, MO calculations and mass fragmentation of the used drugs.^14^

MO calculations show that the total energy values for Fen and Raf are 27.927 kJ mol^-1^ and 28.04 kJ mol^-1^, respectively.

### Determination of purity 

DSC technique can be used for the determination of the purity of Fen and Raf based on the assumption that the impurities will lower the melting point of a pure substance. The melting transition of pure substance should be sharp, but impurities will broaden the melting range and lower the melting point.^15^ Van’t Hoff equation approximately holds and allows the purity value to be calculated as follow:

T_f_ = T_0_ - [(RT_0_^2^ x/ΔH_f_). 1/F]

Where T_f_ is the melting temperature of the sample, T_0_ is the melting point of pure substance in Kelvin (K), R is the gas constant, ΔH_f_ is the heat of fusion, F is fraction of sample melted at T_f_, and x is mole fraction of impurities in the original sample.

ESI 1 shows the DSC curves of Fen and Raf, very strong and sharp endothermic peaks appear at 172.65 °C and 231.90°C which may be attributed to the melting of Fen and Raf, respectively. Table 1 shows the melting point values of these drugs using DTA, DSC and melting point apparatus which agree with those obtained by the reported methods.

**Table 1:** Melting point and purity values of Fen and Raf.

**Drug**	**Melting point (** ^o^ **C)**				**Degree of purity (%)**	
	**DTA method**	Melting point apparatus*	**DSC Method**	**Reported method**	**DSC**	**Reported method**
**Fen**	230.69	231.5	231.90	233^9^	99.17%	99.00%
**Raf**	173.88	172.8	172.65	168-170^3^	99.60%	99.40%^9^

*Stuart SMP 30.

The purity values of Fen (99.17%) and Raf (‏99.60%) are compatible with the data obtained using the reported methods: Fen (99.00%)^1^ and Raf (‏99.40%) ^9^.

## Conclusion

The thermal behavior of Fen and Raf was studied as the first trial in this work using different thermal analysis techniques. The results were confirmed using MO calculations to obtain the correct path way of thermal decomposition of these drugs.

The melting point values obtained by DTA and DSC confirm the precision of these techniques when compared with the reported methods. Therefore these techniques can be used as the identification tools for these drugs based on their melting points. The purity values of Fen and Raf using DSC technique are in agreement with that obtained by the reported methods. Thus, this work reflects the importance of thermal analysis techniques for the quality control of drugs.

## Acknowledgments

The authors would like to express their gratitude to the National Organization for Drug Control and Research (NODCAR, Egypt) and Department of Analytical Chemistry at Faculty of Pharmacy (Cairo University, Egypt) for providing instruments and the means necessary to accomplish this work.

## Ethical Issues

Not applicable.

## Conflict of Interest

The authors declare no conflict of interests.
